# Development of non-invasive ventilation treatment practice for patients with chronic obstructive pulmonary disease:Results from a participatory research project

**DOI:** 10.1177/2050312117739785

**Published:** 2017-11-10

**Authors:** Helle M Christensen, Ingrid L Titlestad, Lotte Huniche

**Affiliations:** 1 Institute of Clinical Research, University of Southern Denmark, Odense, Denmark; 2 Department of Respiratory Medicine, Odense University Hospital, Odense, Denmark; 3 Institute of Public Health, The Research unit of User Perspectives, University of Southern Denmark, Odense, Denmark

**Keywords:** Non-invasive ventilation, chronic obstructive pulmonary disease, treatment practice, participatory research, user perspectives, critical psychology

## Abstract

**Objectives:**

Non-invasive ventilation treatment for patients with acute exacerbation of chronic obstructive pulmonary disease is well documented. Communication with patients during treatment is inhibited because of the mask, the noise from the machine and patient distress. Assessing life expectancy and identifying end-stage chronic obstructive pulmonary disease posed difficulties and caused doubts concerning initiation and continuation of non-invasive ventilation as life-sustaining treatment. Health professionals expressed a need for knowledge of patients’ perspectives and attitude towards non-invasive ventilation.

**Methods:**

The study adheres to principles of Critical psychological practice research. Data on patients’ and health professionals’ perspectives were obtained from observations from the ward and semi-structured interviews with 16 patients. A group of health professionals was set up to form a co-researcher group. The co-researcher group described and analysed treatment practice at the department, drawing on research literature, results from observations and patients’ interviews.

**Results:**

Interviews revealed that 15 patients evaluated treatment with non-invasive ventilation positively, although 13 had experienced fear and 14 discomfort during treatment. The co-researcher group described health professionals’ perspectives and analysed treatment practice based on data from patients’ perspectives developing new management strategies in clinical practice with non-invasive ventilation.

**Conclusion:**

The participatory approach enabled continuous and complementary development of knowledge and treatment practice. The investigation of patient perspectives was particularly productive in qualifying cooperation among health professionals. The study resulted in preparing, and implementing, new clinical strategies.

## Introduction

Chronic obstructive pulmonary disease (COPD) is a progressive lung disease. It is a frequent cause of hospitalizations, visits to the general practitioner, extensive use of medication and reduced quality of life (QoL). COPD is characterized by a chronic inflammatory condition leading to progressive airway obstruction and gradually reduced lung function. The symptoms are breathlessness, coughing, wheezing, sputum, frequent respiratory tract infections and exacerbations.^1,2^ The symptoms progress slowly and are blurred; therefore, patients are often diagnosed in the later stages of the disease.^3^ COPD is an important cause of morbidity worldwide and ranks as the fourth most common cause of mortality.^3^ Smoking is the most important factor causing COPD. At least 50% of smokers contract the disease.^4^ After an acute exacerbation of COPD, the prospect of returning to a prior level of function is small.^3,5^

Non-invasive ventilation (NIV) for patients with acute exacerbation of COPD and with respiratory failure is a well-documented treatment used internationally.^6,7^ Standard treatment for patients with acute exacerbation of COPD also includes medical treatment with bronchodilators, corticoids, optional antibiotics and oxygen.^8,9^ The Department of Respiratory Medicine in this study gives NIV in accordance with British Thoracic Society guidelines, based on evidence group A: patients who benefit from NIV should receive as much NIV as possible in the first 24 h. On the second and third day at least 16 and 12 h, respectively, should be given.^10^ A randomized multicenter study shows that supplementation with NIV decreased mortality from 22% to 9%.^6^ The patient’s ability to tolerate the mask, the recommended hours and synchronizing breathing with the NIV machine play a key role in the result of NIV treatment.^10^ Treatment with NIV is administered by a mask that fits tightly around the patient’s nose and mouth. Communication during treatment with NIV is difficult because of the mask and the noise of the machine. It is also made difficult by the patient’s acute condition, which is often characterized by having fluctuating levels of consciousness, fear and a feeling of suffocation.^11,12^

The presence of acute or chronic respiratory failure and treatment with NIV is often evaluated as a predictor of end stage of COPD.^13,14^ Exactly when the patient with COPD is at the end stage of the disease is difficult to determine. There are no exact biomarkers except low lung function and co-morbidity.^15–17^ Therefore, the health professional has to consider the individual’s physical and mental condition in deciding whether to start treatment with NIV. Difficulties in communicating with patients and recurring acute exacerbations of COPD complicate this decision further. Health professionals often experience resistance from patients while fitting the mask and consider it an ethical challenge to start treatment while patients struggle against their efforts. The health professionals involved in NIV treatment had varying approaches to treatment during end-stage COPD, for example, to always treat the patient regardless of stage of the disease, or withhold treatment in a terminal stage. The health professionals agreed that knowledge of patients’ perspectives on treatment was necessary.

Searches in PubMed, CINAHL, PsycINFO and Cochrane reveal few studies on patient experiences and attitudes towards treatment with NIV. Studies by Torheim and Gjengedal,^11^ Sørensen et al.,^18^ Beckert et al.^19^ and Smith et al.^20^ present patients’ experience with NIV, with a focus on describing the approaches which patients use to adapt to NIV such as feeling of regaining control and trusting skilled help from health professionals. Smith et al.^20^ conclude that more research is needed on patients’ daily lives with COPD versus their attitudes to acute exacerbation of COPD, which can give clinicians a better understanding of the patient’s perspective and thereby build up and qualify management strategies for NIV treatment. The studies don’t look further into the attitude towards receiving NIV considering how they relates to, and live their lives with a chronical illness COPD where hospitalization due to acute exacerbation of COPD is a progress of their illness. Former studies on NIV treatment of patients with COPD conclude that there is need for more investigations about the patients’ and their relatives’ experience and attitudes towards NIV treatment.^21–23^

### Aim

1. To investigate user perspectives on health care practice in the hospital concerning NIV treatment. Users defined as patients, relatives and health professionals.
2. To understand how patients with COPD and health professionals experience and evaluate treatment with NIV.
3. To develop new management strategies for NIV treatment of patients with COPD based on patients’, relatives’ and health professionals’ perspectives on treatment.

## Method

The theoretical framework used in the study was critical psychological practice research. Critical psychology is built on historical dialectical materialism^24^ (Table 1). It focuses the investigation of first-person perspectives on the conduct of everyday life and work practices. Its purpose is to understand how individuals participate in social contexts^24–26^ (Table 1). Critical psychological practice research was chosen because our study intends to clarify first-person perspectives on living with COPD and on health professionals’ clinical practice. Simultaneously, the method used investigates and develop practice by involving practitioners in the research process and continuously feeding results back into practice. The purpose is to develop understandings of why and how individuals act, think and feel about NIV from various positions as patients, relatives or professionals. The advantage of this approach was that it revealed how patients and their relatives experienced illness and treatment in the broader context of their everyday lives. This knowledge could in turn be related to difficulties experienced by health professionals during their efforts to treat and care for these patients in a clinical context.^27–31^ Critical psychological practice research aims to develop theory as well as practice in a joint process.

**Table 1.** Definitions of contexts in critical psychology.

Concepts	Specification
Critical psychology	A psychological theory, also termed the science of the subject, which conceptualizes subjectivity as constituted in the historical development of humanity, society and environment. The basic theoretical framework was published in ‘Foundations of Psychology’ by Klaus Holzkamp in 1985.
Societal context	The material, structural and cultural framework in which subjects unfold their lives, that is, individuals live under societal conditions while also maintaining and changing them.
Conditions, meanings and reasons	Objective conditions, their subjective meanings and personal reasons for acting in specific ways are fundamentally interrelated and must be studied in unison, rather than as isolated units with external connections.
First-person perspectives	Knowledge of subjective meaning and personal reasons is formed from first-person perspectives to understand how conditions produce possibilities and constraints in people’s lives. Subjects conduct their everyday lives by constantly producing and reproducing routines as well as flexible responses to daily challenges.
Conduct of everyday life	Subjects conduct their lives in and across contexts throughout their life trajectories.
Personal ability	The extent to which subjects are able to assume and act with possibilities and constraints in their everyday lives.
Participation	Subjects are conceived as fundamentally societal and as participants in and across social contexts with other subjects.

Source: Smith et al.,^20^ Perrin et al.^21^ and Smith et al.^22^

### Design

A co-researcher group (Table 2) was set up comprising the principal researcher (first author) and six selected clinical co-researchers from a Department of Respiratory Medicine in Denmark. The co-research group was selected to represent all health professional groups involved in treatment with NIV. We strove to recruit health professionals with dissimilar bases of experience and in different age groups. The co-research group convened eight times over the course of 12 months. All meetings lasted from 45 to 90 min and were audio-recorded. The principal researcher contributed with perspectives drawn from participant observation of treatment practices at the department and from semi-structured interviews with patients and relatives following hospitalization and treatment with NIV. The co-researcher group described and analysed practice with NIV, looking for possibilities and constraints in the clinical practice, based on the analysis of the patients’ perspectives of treatment. This design was chosen to identify possibilities and constraint in practice, and at the same time develop, explore and implement new knowledge into the clinical practice with NIV. Participant observation was carried out by the principal researcher to obtain an understanding of the daily routines and practices of NIV treatment at the department.^20,21,31^ Semi-structured interviews were carried out to capture perspectives of patients and relatives.

**Table 2.** Participants in the co-researcher group.

Participants	Professional education	Experience Respiratory medicine
1. Principal researcher (first author)	PhD student	10 years
2. Health care professional	Nurse	2 years
3. Health care professional	Nurse	5 years
4. Health care professional	Nurse	Over 10 years
5. Health care professional	Health care worker	Over 10 years
6. Health care professional	Physician	Less than 1 year
7. Health care professional	Physician, assistant professor	Over 10 years

### Patient and relatives recruitment

Patients’ perspective in this study was based on 20 informants: 16 patients with severe COPD treated with NIV at least once during the last 2 years and 4 relatives (Table 3). This study looks further into the attitude towards receiving NIV considering how they relates to, and live their lives with a chronical illness COPD where hospitalization due to acute exacerbation of COPD is a progress of their illness. It was therefore important to interview the patients after some recovering from the acute exacerbation of COPD. The informants were selected from medical records in the Department of Respiratory Medicine. Recruitment was deliberately constructed to include men and women, single and married and in different ages. Inclusion criteria for the patients were (1) verified COPD, (2) hospitalization and treatment with NIV within the previous 2 years because of acute exacerbation of COPD or pneumonia and (3) not hospitalized at the time of the interview and in a stable period. Exclusion criteria were (1) other imminently life-threatening diseases than COPD, for example, lung cancer; (2) drug or alcohol abuse; and (3) not being able to understand study information and signed consent. The initial 12 informants all said the same in the interviews; all pointed out, in different ways, that they would want treatment with NIV in the future if necessary for a chance to survive an exacerbation of COPD or pneumonia. To be certain, this was not just a coincidence; the co-researcher group sought contact with patients with COPD, who had declined treatment with NIV during hospitalization in 2010 and 2011. Five living individuals were found, one did not respond to contact and four were interviewed.

**Table 3.** Characteristics of informants.

Characteristics	
Number of patients/relatives	16/4
Sex of patients (male/female)	6/10
Sex of relatives (male/female)	2/2
Married/single	10/10
Using oxygen at home	8
NIV (once/more than once)	10/6
Age, mean/median (range)	69/70 (47–86) years
Last FEV_1_ (mean/median)	24/20.5%

NIV: non-invasive ventilation; FEV1: Forced Expired Volume in the first second.

All patients were asked for permission to interview one of their relatives, four accepted and their relatives signed an informed consent form before interviews.

### Data generation process

The Department of Respiratory Medicine agreed to host the research project and to assist in recruiting patients and health professionals. A letter was sent to the informants containing written information on the study aims, design and a consent form. Informants were then contacted by telephone. The principal researcher, who had previous experience with interviews, carried out a face-to-face semi-structured interview with each informant at a location of their choice. All interviews took place in the informant’s home. The interview was partially participant-led. The reason was to enable the informants to tell their story and focus on their experience and thoughts about their illness and treatment with NIV. An interview guide was designed to aid in understanding how patients with COPD experienced treatment with NIV. In addition, it helped determine their understanding of the treatment and to establish whether they would consider receiving the treatment again. The interview questions included four main issues: (1) experience of NIV treatment, (2) the meaning of NIV in everyday life with COPD, (3) main concerns about NIV and (4) the impact of living with COPD and their expectations to hospital treatment. The interviews lasted from 45 to 90 min and were audio-recorded. Each informant received a contact telephone number in case there were questions following the interview. None of the informants made contact after the interviews.

### Data analysis

The thematic analyses were inspired by Kvale and Brinkmann^32^ and analysed with a critical psychological approach. Attention was paid to the informant’s conditions, what they meant to them and how they argued about their conduct of everyday life and choices regarding COPD and NIV.^22–24,27^ All interviews were transcribed and read several times by the principal researcher to identify meaningful units in and across the interviews. The analysis was carried out with attention to the patients’ understanding and experience of constraints and possibilities relating to NIV treatment. In addition, the analysis were carried out with a focus on the informant’s conduct of an everyday life with COPD, patients’ experience of options for actions and the meaning of NIV in the patient’s life trajectories. A further analysis of the main themes from the interviews was carried out in the co-researcher group in light of their clinical experiences and understandings, to develop practices related to NIV based on patient perspectives on treatment. An example of the process on how thematic analysis resulted in development of management strategies is exemplified in Table 4. In that way, analysis was ongoing during the interviews, which allowed specific themes to be further explored in later interviews.

**Table 4.** Thematic analysis process.

Patients’ and relatives’ perspectives	Health professionals’ perspectives and analysing user perspective on treatment with NIV	Action and attitude strategies developed
*Understanding resistance towards treatment*		
Patients interview revealed three different scenarios on explaining resistance against treatment: 1. Resistance due to a natural reaction to the feeling of being strangled: P11: I could not even turn myself in bed. They came and pushed it down over my head. And it was so terrible. I thought I would die. But it was as if they intentionally tried to choke you. It was as if they just killed you. 2. No resistance even though NIV is not experienced as being comfortable: P13: Because I’ve always said to myself, there are none of them who would do something to me that is not necessary. So … That’s life. It’s like when they come and sticks me, just poke I say; it doesn’t bother me at all. 3. Expecting treatment with NIV despite showing resistance: P13: Yes then I would like to have it on, to rescue whatever it can rescue, if that’s possible, right? Yes in that way, then it has been tried, what can be done, and then that is that.	Health professionals had overall two approaches on how to react to resistance from patients when initiating NIV: 1. To treat no matter what, keeping in mind the patients were cerebrally and mentally affected by the high blood-CO_2_ levels and the acute situation: C: Yes but I don’t think, you can say they have their own determination if they are not in their right mind. If they have a pH of 7.15, then you are also not able to carry out their own determination. 2. Considering not treating because of the patient’s resistance, interpreting it to mean ‘I don’t want treatment with NIV’, especially when the patient is in the end stage of the disease.	Decision to train new staff to focus on the patients’ perspective; the study showed that knowledge about patients wanted treatment with NIV even though showing resistance had a major impact on how health professionals could cope with the ethical dilemmas when treating with NIV. Interdisciplinary joint ward rounds to ensure shared approach and attitude towards treatment based on the individual patient perspective. Establishment of a room at the department, reserved for four patients receiving NIV. To make it possible for the health professionals to stay in the room for a longer time while starting treatment with NIV, to help patients to cope with the mask and to open possibilities to provide advanced nursing when needed.
*Physical discomfort or relief*		
Patients had two ways of describing the experience of physical impact of NIV: 1. As a help and a relief: P1: I could feel it helped, it was a relief, I think it was good, I think it helped. R5: I think that it was great that I could see how well the body was moving. 2. As a discomfort: P5: I was about to get claustrophobia from it I think. I simply could not handle it anymore, it’s like that, and I don’t know why. Whether it’s because you get trapped and you feel you aren’t doing well. Whether that is what makes it, I cannot give you an explanation. I do not know why it should be so tight. P11: I had to go on, and I had to bring the number up and I said, I simply cannot breathe in it. I felt that it was just as if one would strangle you.	Health professionals hold the view that most patients experienced treatment as uncomfortable due to the tightness of the mask. They found that a few patients experienced the treatment as a relief due to the breathing assistance and reducing feeling of being strangled. Nursing care was expressed as being important in the beginning phase. Being experienced in adjusting the mask and NIV machine was considered an important factor in helping the patient to cope: C: I was thinking, those statements may ease the process for the people who think ‘now I have to put the mask on’ when there are statements from the patients, that say even though I resist, I still actually want it (NIV).	Increased focus on physical presence as well as guiding the patient to cooperate with the NIV mask in the initiating phase. To help the patient in coping with the mask, introduce the possibility of being treated with NIV at an earlier stage of the disease and explain why the treatment is important. Focus on special training of new staff, and updating old staff with the new insights during a workshop.
*Coping with anxiety*		
All patients described anxiety during the treatment: P2: Yes, but I was afraid. I simply could not … All the nerves were simply gone. There was nothing left. And then fear comes into it and makes it much worse. Then you panic and then … P16: If they had come quietly and said we’ll give you just a little sedative before you get the mask on and explained to me what the mask was about. Yes sometimes you do it, well and sometimes you have to, I know it is about the breathing, but the fear is worse.	Health professional reported that all patients displayed anxiety, and there were different suggestions on how to help the patient to cope: C: I actually think we need to, at the end-stage COPD, have some sedative, which we do not have. C: As they say themselves, I’m not afraid of dying but I am afraid of the time before. I’m afraid of being strangled, as the terminally ill cancer patients says, but our severely ill COPD patient says the same. P: If you inform them (patients) in the beginning, and that is actually, why the nurse is to do that, because they have a function close to the patient, then you can spend some of the time in the beginning and then you don’t have to spend so much time later.	Increased focus on being clearly present to the patient, to help cope with anxiety when initiating treatment with NIV. Establishment of one room at the department for patients being treated with NIV also enabled the nurses to be able to stay closer to the patients, made them available. Decisions regarding use of sedatives should be made during the daytime with the patient. A specialized trained staff should make these decisions. Using the word ‘lung palliative’ to focus on special caring needs for patients in the end state of COPD. Information to the patient being treated with NIV must be repeated often due to cerebral impact.
*COPD in everyday life*		
Patients were telling about how they coped with COPD in their conduct of everyday life. Looking at COPD as a basic condition: P4: I do cough a little and so on, but otherwise I don’t think I kind of have particularly poor breathing. No I don’t think I have had that. But it’s got something to do with fitness and stuff so, no, I’m walking well – I cut the grass, and I take care of the garden and things like that, I do – it just takes a little longer, but then never mind. But it does, it takes longer. P16: No I don’t, when I have finished my morning toilette and I have caught my breath again, I’m sitting here getting my breakfast. Then I don’t think at that I’m ill, then I feel good again. How everyday life with COPD has an impact on how patients experience the reasons for hospitalization. P11: Well, yes, and when my COPD is bad, like now, it is due to a cold, that’s how it starts. I don’t consider COPD as a huge problem for me; it may be a mistake but …	Health professionals worked with the patients’ understanding of their disease: C: Because it’s the patient who holds all the cards in their hand in relation to what I want with my life; what they have, and how I would like to leave this life here. C: It was just one of those things she (the patient) said, that they are hospitalized, and they get diagnosed, and then they say, oh my God, now I am suddenly ill. No, you have been ill for many years. C: I think it is the same as we learn in TeleKOL (Study), because they don’t look at their disease as something they can die of, rather as a part of their everyday life, it’s a natural part of their lives.	The nursing care of patients with COPD is to be based on the knowledge that they want the treatment even though they show resistance. Focus on this especially in training new staff in the department. Instead of using terminal aspects in treatment, a workshop with the health professionals contributed to naming aspects of treatment interventions for lung palliation. The change of words should draw attention to a focus on patients’ everyday life with a disease considered as a basic living condition, and therefore rarely realized by the patient.

NIV: non-invasive ventilation; COPD: chronic obstructive pulmonary disease.

### Ethical considerations

The study is approved by the Danish Data Protection Agency (J.nr. 2011-41-6218) and conducted in conformity with the Helsinki declaration. All the informants’ names in this article are pseudonyms to ensure anonymity.

## Findings

Four main themes emerged through the analysis: understanding resistance towards treatment, physical discomfort or relief, coping with anxiety and future treatment with NIV. All these themes interact with one another but are presented separately in the following presentation.

### Understanding resistance towards treatment

Data from health professionals describing their experiences from the clinical practice with NIV showed different understandings of the experience of the patient’s resistance to the treatment: when placing the NIV mask over patient’s mouth and nose at the beginning of the NIV treatment, health professionals often experienced patient resistance. Communication during treatment was experienced as difficult due to the tight fit of the mask and the acute condition. It was further exacerbated by the patient’s fear, impaired consciousness and feelings of suffocation. Due to difficulties in communicating, health professionals interpreted the resistance as an expression of not wanting treatment. When the patient was in the end stage of COPD, ethical considerations whether to override the resistance or to terminate treatment were often-discussed issues. To develop management strategies in these situations, there was a need to understand patients’ perspectives on treatment with NIV, that is, how patients with COPD understood and related to treatment. When interviewed at home after discharge from hospital, some patients did not remember if they had struggled against the mask. Most patients expressed anxiety due to the unfamiliar technology of the NIV treatment. They explained that their resistance was due to not knowing enough about the treatment, not being able to take in and understand what the NIV machine did and what it was good for:

I simply did not know what it was. I thought I hadn’t got any information about it – also perhaps because I had been too far away – I do not know. I probably was. Since I lacked some information afterwards, I think. (P7)

Some patients were not aware, until afterwards, that there were no possible alternative treatments. Breathing troubles were also cited in explaining their resistance. During an exacerbation of COPD, their only thought was, to keep working on being able to breathe, keeping the airway as free as possible. Feeling strangled by the NIV mask caused anxiety. Not being able to think straight, made them focus on breathing as a main issue. The patients explained that they were often not able to see their conditions in a larger perspective in this situation:

I could not even turn myself in bed. They came and pushed it down over my head. And it was so terrible. I thought I would die. It was – it was as if they intentionally tried to choke you. It was as if they just killed you. (P11)

Health professionals expressed two approaches to patient resistance: One was to interpret resistance as an expression of not wanting NIV treatment. The other was treating to help the patient through the acute situation. In this instance, the resistance was considered a natural consequence of hypoxaemia. The co-researcher group all agreed on the importance of developing management strategies in NIV practice based on patient views and understandings of the treatment. Understanding what the patients’ perspective was based on when hospitalized became an important issue in further interviews. It appeared in the interviews that 15 out of 16 patients and 4 relatives all expected and wanted to be treated with NIV despite displays of resistance. The patients explained resistance as a natural reaction to not being able to breathe and not having the necessary knowledge and experience with NIV treatment.

### Physical discomfort or relief

In general, all the patients said that NIV was an uncomfortable treatment due to the tight mask pushing air in and out, demanding the patient to cooperate with the machine. Four patients experienced a feeling of claustrophobia due to not being able to move from the bed and the tight mask. Some patients experienced the mask as a relief as the feeling of being choked receded. One patient explained,

You can try to have someone to pull a pillow over your mouth, then you can get a sense of it, I think that’s probably the best way to … (P6)

Two patients felt NIV treatment as a relief and attached hope to the fact that something was being done about their condition. All relatives had the same experience. The machine was new to clinical practice and not well known among patients with COPD. The informants expressed difficulties in collaborating with the machine due to not knowing the purpose of treatment and thinking there might be other possibilities. Both patients and health professionals experienced large amount of noise from the machine. Combined with the breathing problems, it was difficult to communicate and understand information, especially at the onset of the treatment. Informants, who had received the treatment more than once, said that knowing what it all was about, made it much easier to cooperate in the acute situation. This study reveals that experience with treatment and the machine eases the patients’ anxiety and improves cooperation.

### Coping with anxiety

All patients in this study talked about anxiety: anxiety of being choked, being alone with new technology, lack of predictability. They asked themselves; when will you be able to have something to drink? When will the staff be back? How long will the treatment with the mask go on?

It is an uncertainty that comes from being strapped into something that does something for you, which your body does not really want. It’s like someone puts something on your head, that does something by itself, that your body doesn’t want … you are all alone in the world, nobody whispering in your ear: ‘Try to follow the breathing’. Nobody sits beside you. (P6)

Some of the informants expressed a need for human closeness, a feeling of a hand on the arm or just knowing they were not alone, that staff had an eye on them, or knowing that staff would be back at certain intervals. Knowing that you are not alone in coping and cooperating with new technology helps decrease anxiety:

I did not get any support; they just said ‘so and so’ that’s just not me. If they had given me a little sedative. A touch on the hand or something, yes, that’s a big help. Then you’re like, oh yeah, they have an eye on you … you just have to take it slow, they look after you. I think that was what I missed the most. I was left to myself in the machine. It was like I felt like I was all alone in the world and then you become afraid of what happens next, what happens now. (14K)

Decreased anxiety enabled patients to put more effort in cooperating and tolerating the NIV treatment. The person interviewed last had doubts about treatment with NIV, not because she did not want treatment, but because she would rather have treatment in a respirator. She explained the treatment made her feel claustrophobic; she found it difficult to tolerate the mask because of the tightness and the anxiety that it produced. To cope with the anxiety, she explained that she needed to know she was not alone and could contact health professionals regularly. She said that she would have been able to cope with the claustrophobia, if she had known from the start when she could have a small break to breathe, and water to drink. She said all she needed was 5 min every 4 h. The patient also said it would help to know from the beginning of the treatment, whether it was possible to get anxiolytic medication during the night. She needed to know her possibilities before night-time, to be able to cope and work together with the machine. If these aspects could be part of the treatment, she would want NIV in the future. Health professionals expressed that communication during treatment was perceived as difficult because of the tight fit of the mask and the acute condition. Patient’s fear, impaired consciousness and feelings of suffocation also made communications difficult.

### Future treatment with NIV

Interviews revealed that Chance of survival was voiced by 15 out of 16 patients (supported by 4 relatives) as a reason to accept NIV treatment in future. The initial 12 informants all pointed out, in different ways, that they would want treatment with NIV in the future if necessary for a chance to survive an exacerbation of COPD or pneumonia. Three informants who previously had declined NIV treatment during hospitalization had changed their opinion about treatment with NIV after being discharged from hospital. All the patients, with the exception of number 16, expected the best possible treatment. They also expected health professionals to be ‘the ones who knew best’ with a knowledge of the optimal and available treatments in the acute situation:

I expect that you know more about this than I do, and that you do, whatever you have to do. I have always trusted … what is it you call it, the authorities. If you think it might work, then I think it is okay. Because for sure, I don’t know as much as you do, because you see more of my kind in here. (P4)

All patients stated that they, in connection with their hospitalization, expected to be treated if there was a hope of survival. Through the interviews, it was made clear the informant’s conduct of everyday life with COPD had a great impact on, what expectations the informant had towards hospitalization due to breathing problems. Patients’ interviews showed they considered living with COPD as a basic condition more than a disease. The event of an acute exacerbation of COPD causing a need for hospitalization was considered by the informants as a result of a cold or pneumonia, a new situation that was well known and normally could be treated. Patients’ interviews revealed that they relied on health professionals being well-educated and able to take the responsibility of starting treatment with NIV, even in the face of patient resistance. Contrary to this, health professionals understood hospitalization and NIV treatment to patients with COPD as a development or worsening of COPD. They therefore understood the resistance against treatment as a way of showing that patients did not want the treatment due to the severe COPD.

## Discussion

### Strengths and weaknesses of the study

A strength of the study was to investigate drawing on user perspectives. It emerged to play a crucial role in developing new management strategies to improve the quality in treatment and care to patients with COPD. Another strength was the similarity in the patient’s perspectives on treatment. In all, 15 out of 16 patients wanted treatment with NIV in the future despite resistance in the acute situation. Interviews also revealed that chance of survival was voiced by 15 out of 16 patients (supported by 4 relatives) as a reason to accept NIV treatment in future. The emerging patients’ perspectives and management strategies resulted from the involvement of the co-researchers and could be integrated into daily routines and also further reflected upon during the research process. The joint analysis of the clinical practice with NIV treatment on the ward and the shared data analysis involving both patients and health professionals’ perspectives ensured consistency of results. The co-researcher group confirmed the main themes identified by the researcher in the semi-structured interviews of the patients and their relatives.

Weakness of the study: Given the health needs of COPD patients, it was impossible to involve them more actively in the study. This lack of patient representation (either in the co-researcher group or in a reference group) could be seen as a weakness. To work with a co-researcher group with members from interdisciplinary health professionals required the department to allow time for the meetings. It could only be done with support from the department management and commitment from all staff members. This is a potential weakness of the methodological approach. A potential weakness is also the lack of time from patients’ hospitalization and the semi-structured interview. This time break can cause memory bias. Still this study looks further into the attitude towards receiving NIV considering how they relates to, and live their lives with a chronical illness COPD, where hospitalization due to acute exacerbation of COPD is a progress of their illness. It was therefore important to interview the patients after some recovering from the acute exacerbation of COPD.

### Summary of main findings

Four main themes emerged through the analysis: understanding resistance towards treatment, physical discomfort or relief, coping with anxiety and future treatment with NIV. The patients explained their resistance against NIV during treatment as being due to anxiety, difficulties in coping with NIV and blurred consciousness. Patients and health professionals had different approaches towards hospitalization and treatment. This was clarified through joint analyses in the co-researcher group. Patients considered the hospitalization and the treatment as a new illness and the COPD as a basic condition of their conduct of everyday life. Health professionals considered a patient with severe COPD being hospitalized due to breathing troubles as a continuing progress of COPD. The discrepancies in the approaches towards hospitalization were the main reason for complications in daily management. New management strategies were developed based on these perspectives.

The patients in this study expected health professionals to make enlightened decisions in the acute situation of beginning treatment with NIV. At this point, patients’ consciousness was often blurred due to anxiety, increased CO_2_ levels in the blood and fatigue. Because patients conducted their everyday lives with COPD as a basic condition, the patients did not consider COPD as the reason for hospitalization. Rather, they looked at it as a new event such as a cold or pneumonia, which was usually treatable. This study showed that the conduct of everyday life has a great impact on the expectations patients with COPD have to hospitalization due to breathing troubles. Patients’ conduct of everyday life with COPD as a basic condition rather than an illness and how it influenced their expectations to hospitalizations was also confirmed in the study by Pinnock et al.^15^ Pinnock et al. investigated patients’ attitudes towards living with COPD. COPD was considered as a basic condition in their lives, based on the slow development of the disease, without a start date. The study results by Pinnock et al.^15^ were included in analyses of patients’ perspective and supported the findings. Our results showed that focusing on user perspectives in health care practices consisting of NIV treatment in a hospital ward, and defining users as patients, relatives and health professionals, contributed to new knowledge that was useful in developing management strategies for this treatment. It was therefore important to use the knowledge of these patients’ perspectives on treatment in developing management strategies for NIV. This practice enabled exploration and development of new clinical strategies based on patient and health care professional’s perspectives on treating patients with COPD with NIV.

### Results in relation to other studies

The results from this study are comparable to a study by Pinnock et al.^15^ on how patients with COPD live their everyday lives, in and across different contexts, experiencing COPD as a condition of life rather than as illness: ‘Not so much an illness, more a way of life’.^15^ This study showed that a lack of knowledge of patient perspectives on treatment was problematic. It could cause major problems in everyday clinical practice in a hospital ward, when starting up treatment with NIV to patients in the end stage of the disease. The study by Gore et al.^16^ comparing how patients with lung cancer and COPD are treated shows that these patients’ needs are much alike, because of a communality of symptoms. However, care for patients with COPD is deficient because of the invisibility of the disease and the impact on how patients live their everyday lives. Problems due to the NIV mask were investigated in the study by Sørensen et al.^18^ and Torheim and Gjengedal.^11^ The studies reported that patients describe anxiety as a major aspect in coping with NIV treatment.

The patients were interviewed up to 2 years after treatment which is a rather long duration. It may not necessarily reflect the absolute experience during the treatment itself due to memory relapse. This study looks further into the attitude towards receiving NIV considering how they relates to, and live their lives with a chronical illness COPD, where hospitalization due to acute exacerbation of COPD is a progress of their illness. The focus was to interview the patients after some recovering from the acute exacerbation of COPD. All the informant could in detail describe and relate to their hospitalization and treatment with NIV.

This study revealed differences in patients’ and health care professionals’ perspectives on treatment and their expectations concerning the hospitalization (Table 5). It highlighted the importance of exploring and drawing on patient’s perspectives in other areas of treatments and care to patients with COPD.

**Table 5.** What this study adds.

What was known	What this study adds
There is solid evidence for using NIV as treatment for patients with COPD in exacerbation.	Health professionals experience ethical challenges when starting treatment with patients who resist. This is particularly true of end-stage patients.
Special skills are important for nursing patients in NIV treatment. This is due to patient difficulties in connection with the NIV machine.	Special nursing skills are required when starting NIV treatment with COPD patients. Skills comprising technical knowledge, helping patients cope with anxiety, physical presence, predictability in the care process, information about the NIV machine and help to learn how to cooperate with it.
Patient often shows resistance when starting up NIV treatment.	Patients explain resistance as an automatic response to experiencing breathing troubles and being affected by the emergency. It feels unnatural to have something plastic fitted tightly over nose and mouth. In a patient’s conduct of everyday life, COPD is more a condition of life than an illness. This means that hospitalization is experienced as a new event: A cold, the flu or pneumonia which can usually be treated. Therefore, they expect treatment when hospitalized and they expect health professionals to do whatever they can, in the hope of getting through the acute stage.

NIV: non-invasive ventilation; COPD: chronic obstructive pulmonary disease.
